# Supplementary material for: Bioinformatic Approach to Identify Positive Prognostic TGFB2-Dependent and Negative Prognostic TGFB2-Independent Biomarkers for Breast Cancers
Source: Int J Mol Sci. 2025 Nov 29;26(23):11580. doi: 10.3390/ijms262311580 (PMC12692321; doi:10.3390/ijms262311580)
Supplement: Supplementary file 1 [file ijms-26-11580-s001.zip › ijms-3887601-supplementary.pdf]

Supplementary methods.

Oncotelic Chatbot Libraries

PubMed abstracts were downloaded as text documents for processing using the Oncotelic Chatbot technologies. Each abstract was then indexed into our Quadrant database (aided by Puppeteer 24.10.0), embedded, and transformed (using LangChain-OpenAI 0.6.16 and OpenAI 5.20.1) into a vector of numbers that captures semantic similarity between text elements (tokens). The embedding transforms all abstracts to the same vector "embedding" space. It has been trained to minimize the distance (in vector space) between any pair of abstracts to the extent that they are semantically similar. In the question-answering session, the user query was transformed into an embedding vector. Then an appropriate similarity measure (eg. Cosine distance) was used to identify the embedded abstract vectors closest to the embedded query vector: the abstracts corresponding to these matching vectors were then fed to the question-answering model (in the form of context), along with the query, to arrive at an answer to the query.

The user interface is powered by the React framework, an open-source and flexible JavaScript library for developing powerful front-end interfaces (https://react.dev/ accessed March 25, 2024). Additionally, we utilized the @mui/material library (https://mui.com, accessed March 25, 2024) for the interface's design aspects, aiming to follow the Material Design guidelines closely. Serving the front end was Node.Js (https://nodejs.org/en Accessed 25/03/2024). Node.js libraries included in the project: @adobe/pdfservices-node-sdk 3.4.2@aws-sdk/client-s3 3.412.0, @langchain/community 0.3.57, @mui/base 5.0.0-beta.18, @mui/icons-material 5.11.16, @mui/material 5.15.20, @mui/styled-engine-sc 5.12.0, @mui/x-date-pickers 6.15.0, @qdrant/js-client-rest 1.15.0, pdf-img-convert 1.2.1, puppeteer 24.10.0, react 18.2.0, sequelize 6.37.7

Oncotelic Chatbot Methods

We gathered abstracts using keyword searches on the PubMed database. We then proceeded to execute two procedures: 1. Direct LLM querying; and 2. Semantically filtered LLM querying.

The direct LLM query procedure was executed as follows: given the initial set of abstracts and a specific question, we sent each abstract to an LLM, along with the question, and received a response. For each abstract, we evaluated the response and rejected those abstracts that did not meet the criteria. For each surviving abstract, we further evaluated the abstract text itself and rejected those abstracts that did not meet the criteria.

The semantically filtered LLM query procedure was executed as follows: we parsed each abstract and split it into one or more text fragments. We then converted each fragment to a vector in a 3072-dimensional "embedding" space. We then also converted the question to a vector in the same embedding space. The embedding space has the property that the distance between vectors is inversely proportional to the semantic similarity between the fragments to which they correspond. For each fragment, we thus evaluated the semantic similarity between the question and the fragment by thresholding the distance (strictly: the cosine distance) between their corresponding vectors. We rejected those fragments whose distance exceeded the threshold. We then sent each surviving fragment to an LLM, along with the question, and received a response. For each fragment, we evaluated the response and rejected those abstracts for which no fragments met the criteria. For each surviving abstract, we further evaluated the abstract text itself and rejected those abstracts that did not meet the criteria.

Both direct LLM querying and semantically filtered LLM querying were executed for each of the following sets of questions. The final set of abstracts, which were included in the analysis, comprised the union of the set of abstracts that survived for each question and for each query method (see flowchart).

Flowchart for Oncotelic Chatbot use.

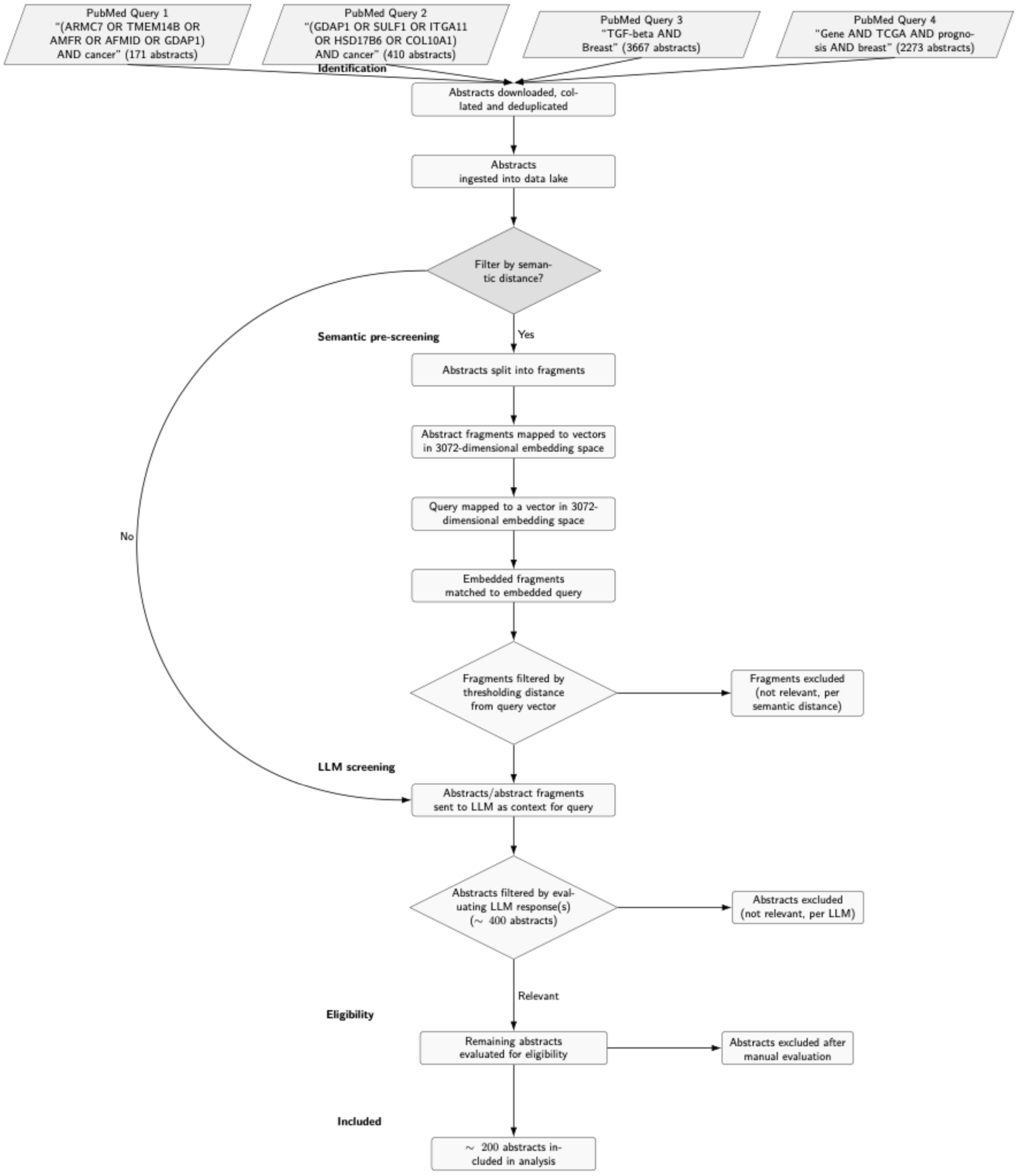

Perplexity-pro follow-up to further investigate the PubMed abstracts.

Perplexity AI Pro (Academic Sources Only Mode) was employed to identify and access relevant academic sources through the following process. The gene or protein identified from the modelling results was combined with its associated disease context and biomarker classification using standardized names or identifiers. Focused prompts concerning gene function, biomarker status, and disease associations were prepared, using varied phrasings of each question (listed below). These prompts were entered into Perplexity AI Pro in separate threads, typically generating around seventy cited references for each gene report, organized by publication date and lead author.

Duplicate and non-peer-reviewed references were subsequently filtered out. Each remaining reference was individually examined and validated, with irrelevant sources removed. This process generally yielded between five and ten directly relevant references. All validated references, including full metadata (authors, title, journal, year, DOI/PMID, and URL), were archived for detailed study and used to support the development of the paper. Only information explicitly substantiated by these cited sources was included in the written text.

The draft text, containing in-text citations, was submitted to the lead author for assessment of citation accuracy, relevance, academic quality, and informational value. This procedure has been formalized as a standard operating protocol for employing large language model (LLM) artificial intelligence in secondary research. The specific prompts used for this study are listed below.

Initial General prompts for Oncotelic and Perplexity responses

1. Provide an overview of High-Grade Serous Ovarian Carcinoma
2. Summarize prognosis and survival outcomes for ovarian cancers
3. Summarize factors influencing long-term survival
4. Summarize genetic and molecular prognostic markers
5. What are the standard treatments and challenges
6. What are the roles of TGF-β in the Tumor Microenvironment
7. Summarize molecular profiling and personalized therapy approaches
8. Summarize the use of prognostic models and gene signatures for risk stratification
9. Summarize the impact of TGFB2 specifically in tumors.

Initial Gene-specific prompts for Oncotelic and Perplexity responses

10. What is the role of [gene] in human physiology?
11. What is the role of [gene] in genetics and biochemical mechanisms that affect physiological function?
12. What is the role of [gene] in cancers?
13. Summarise findings that explicitly state the role of [gene] in cancer progression, cancer resistance, tumour physiology and patient survival. Focus on humans and mouse genetic model systems.
14. What evidence is there that [gene] has a role in breast cancer?
15. Summarise findings that explicitly state the role of [gene] in breast cancer progression, cancer resistance, tumour physiology and patient survival. Focus on humans and mouse genetic model systems.
16. What evidence is there that [gene] interacts with TGFB pathways?
17. Provide a thorough and comprehensive response, highlighting the interaction between [gene] and the TGFB pathways, detailing any genes that might connect [gene] to TGFB pathways.

These were questions applied across all the marker genes. More specific questions that arose from the core set of questions were further investigated.

The Chatbot technology allows users to conduct question-and-answer sessions to refine the references to be used in manuscript preparation. This was not a systematic review, but an expansion of keywords used for PubMed searches by having an interactive console to ask further questions to refine the screen, and we used other databases (Perplexity AI) to find articles that may not have been deposited in PubMed. The chatbots were not used for any validation of the results, formulating a hypothesis, or writing the paper. The bioinformatic analyses drove the discovery of all biomarkers in the manuscript.

Supplementary Figures.

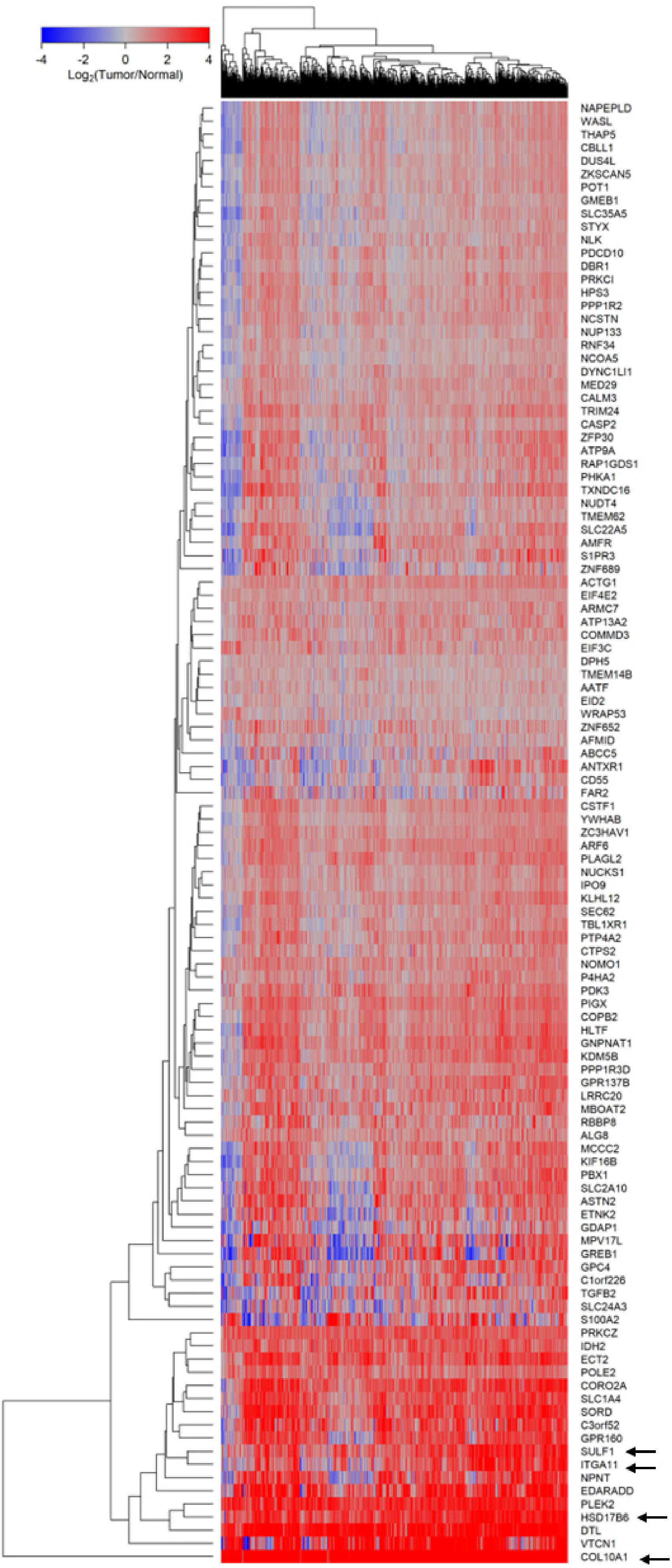

-4
-2
0
2
4
Log2(Tumor/Normal)
NAPEPLD
WASL
THAP5
CBLL1
DUS4L
ZKSCAN5
POT1
GMEB1
SLC35A5
STYX
NLK
PDCD10
DBR1
PRKCI
HPS3
PPP1R2
NCSTN
NUP133
RNF34
NCOA5
DYNC1LI1
MED29
CALM3
TRIM24
CASP2
ZFP30
ATP9A
RAP1GDS1
PHKA1
TXNDC16
NUDT4
TMEM62
SLC22A5
AMFR
S1PR3
ZNF689
ACTG1
EIF4E2
ARMC7
ATP13A2
COMMD3
EIF3C
DPH5
TMEM14B
AATF
EID2
WRAP53
ZNF652
AFMID
ABCC5
ANTXR1
CD55
FAR2
CSTF1
YWHAB
ZC3HAV1
ARF6
PLAGL2
NUCKS1
IPO9
KLHL12
SEC62
TBL1XR1
PTP4A2
CTPS2
NOMO1
P4HA2
PDK3
PIGX
COPB2
HLTF
GNPNAT1
KDM5B
PPP1R3D
GPR137B
LRRC20
MBOAT2
RBBP8
ALG8
MCCC2
KIF16B
PBX1
SLC2A10
ASTN2
ETNK2
GDAP1
MPV17L
GREB1
GPC4
C1orf226
TGFB2
SLC24A3
S100A2
PRKCZ
IDH2
ECT2
POLE2
CORO2A
SLC1A4
SORD
C3orf52
GPR160
SULF1
ITGA11
NPNT
EDARADD
PLEK2
HSD17B6
DTL
VTCN1
COL10A1

Figure S1. Upregulated genes in tumor tissues with a significant statistical interaction of *TGFB2* and the marker gene mRNA. Multivariate analyses utilized the Cox proportional hazards model to assess the individual effects of *TGFB2* and marker gene (Gene2) mRNA expression levels. 111 genes with a significant effect of the *TGFB2* by Gene2 interaction term exhibited significant upregulation in tumor tissues ($p < 0.0001$, FDR < 0.001). The cluster figure shows the mean-centered tumor tissue expression compared to normal tissues for 11 out of the 111 genes examined (log2 (tumor/normal TPM), color-coded from blue to red, indicating a decrease to an increase in expression in tumor tissues, respectively). Arrows depict a cluster of genes with the highest fold change in tumor tissues exhibiting hazard ratio calculations for *TGFB2* mRNA expression ($p < 0.05$), Gene2 expression ($p < 0.05$) AND *TGFB2* by Gene 2 interaction terms ($p<0.05$).

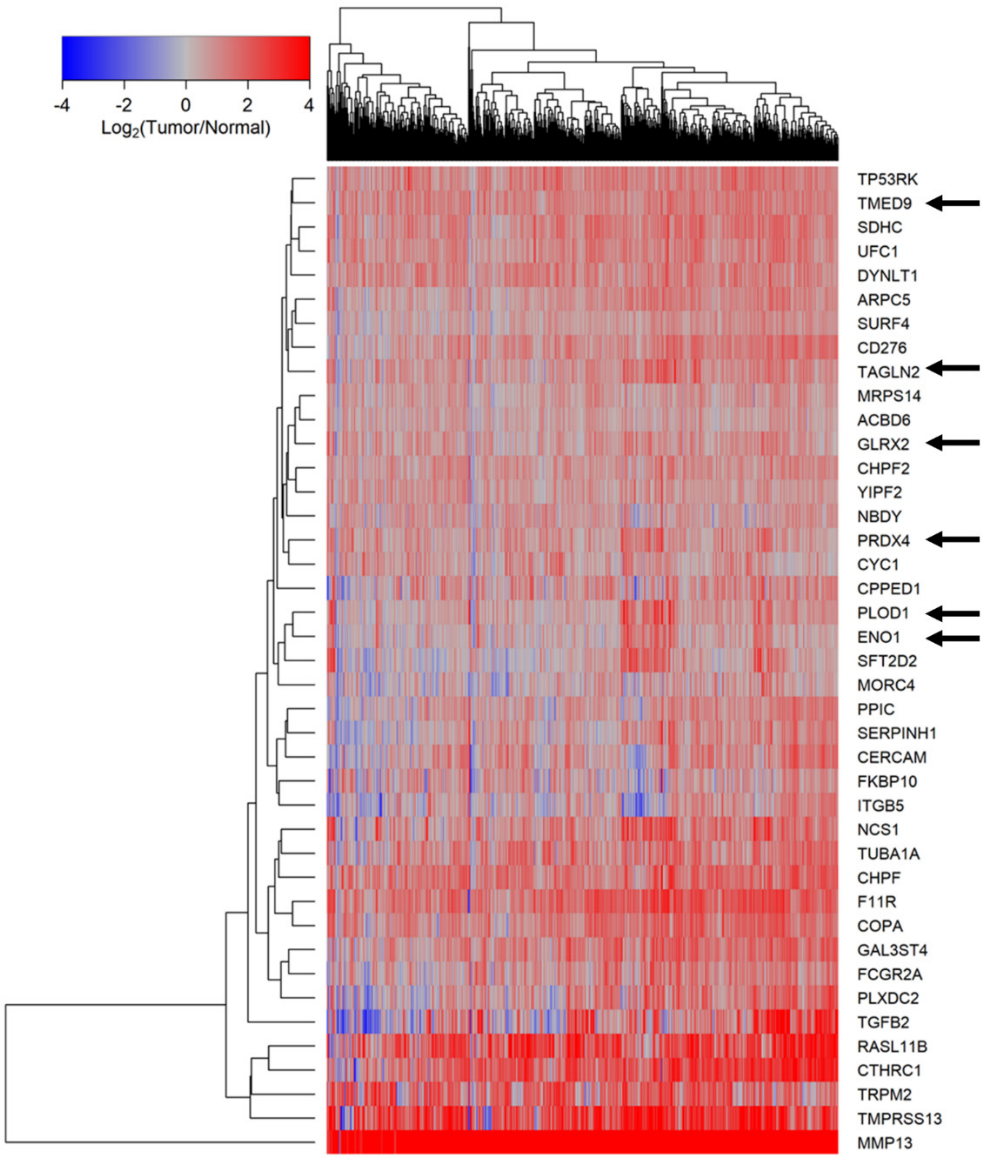

Figure S2. Negative prognostic markers identified from the multivariate Cox proportional hazards model were upregulated in breast cancer tumor tissues. A multivariate analysis utilizing the Cox proportional hazards model to assess the individual effects of *TGFB2* and Gene2 marker mRNA expression levels to identify marker genes predicting worse prognosis, not dependent on *TGFB2* mRNA expression (*p*-val *TGFB2* mRNA > 0.1 AND *p*-val *TGFB2* x Gene2 interaction > 0.1 AND *p*-val Gene2 < 0.05 & HR Gene2 > 1). The cluster figure depicts 41 genes significantly upregulated in tumor tissues (*p* <0.0001, Fold Change > 1, $\log_2$ (TPM) Expression in tumor tissue > 2) for 786 evaluable patients. We cross-referenced the 41 genes identified in the TCGA dataset with those reported in the KM plotter database to narrow down the potential list of prognostic markers using more stringent criteria, comparing only median cut-off values for high versus low-expressing patient sub-sets. The black arrows indicate the expression values of six significantly upregulated genes in breast cancer tumor tissues, as cross-referenced in both TCGA and KMplotter datasets.

Table S1. Multivariate Cox proportional hazards ratio calculations for *TGFB2* interaction genes upregulated in tumor tissues.

	**Gene 2 mRNA**		***TGFB2* mRNA**		**Chemotherapy only**		**Age at Diagnosis**		***TGFB2* by Gene 2 Interaction**	
**Gene 2**	**HR(95% CI)**	***p-val***	**HR(95% CI)**	***p-val***	**HR(95% CI)**	***p-val***	**HR(95% CI)**	***p-val***	**HR(95% CI)**	***p-val***
HSD17B6	1.43 (1.16-1.76)	0.001	0.61 (0.37-0.99)	0.045	1.57 (0.72-3.43)	0.26	1.03 (1-1.05)	0.019	1.47 (1.03-2.1)	0.036
ITGA11	1.41 (1.17-1.69)	<0.001	0.56 (0.33-0.95)	0.032	1.62 (0.74-3.55)	0.229	1.03 (1-1.05)	0.018	1.5 (1.06-2.12)	0.023
GPC4	1.37 (1.05-1.78)	0.019	0.61 (0.38-0.98)	0.042	1.57 (0.72-3.42)	0.26	1.02 (1-1.04)	0.044	1.54 (1.07-2.22)	0.02
COL10A1	1.37 (1.13-1.66)	0.001	0.56 (0.33-0.93)	0.027	1.56 (0.71-3.42)	0.268	1.03 (1-1.05)	0.017	1.39 (1.07-1.79)	0.012
ANTXR1	1.36 (1.16-1.6)	<0.001	0.58 (0.36-0.95)	0.029	1.55 (0.7-3.4)	0.277	1.03 (1.01-1.05)	0.014	1.35 (1.01-1.81)	0.043
SULF1	1.22 (1-1.49)	0.046	0.61 (0.38-0.98)	0.039	1.51 (0.69-3.31)	0.302	1.03 (1.01-1.05)	0.014	1.35 (1.05-1.73)	0.021
ARMC7	0.62 (0.42-0.93)	0.021	0.5 (0.26-0.96)	0.038	1.5 (0.68-3.29)	0.313	1.02 (1-1.04)	0.036	0.47 (0.23-0.93)	0.03
TMEM14B	0.49 (0.28-0.86)	0.013	0.39 (0.17-0.9)	0.027	1.53 (0.7-3.34)	0.287	1.02 (1-1.04)	0.038	0.35 (0.13-0.94)	0.038
AMFR	0.42 (0.22-0.82)	0.011	0.39 (0.17-0.91)	0.03	1.4 (0.64-3.06)	0.393	1.02 (1-1.04)	0.044	0.32 (0.11-0.96)	0.041
AFMID	0.22 (0.08-0.67)	0.007	0.45 (0.22-0.94)	0.035	1.32 (0.6-2.92)	0.489	1.02 (1-1.05)	0.027	0.1 (0.02-0.62)	0.013
GDAP1	0.02 (0-0.56)	0.022	0.19 (0.04-0.78)	0.022	1.52 (0.7-3.32)	0.289	1.02 (1-1.04)	0.038	0 (0-0.39)	0.022

	**Basal subtype**		**HER2+ subtype**		**Luminal A subtype**		**Luminal B subtype**		**Normal subtype**	
**Gene 2**	**HR(95% CI)**	***p-val***	**HR(95% CI)**	***p-val***	**HR(95% CI)**	***p-val***	**HR(95% CI)**	***p-val***	**HR(95% CI)**	***p-val***
HSD17B6	0.58 (0.24-1.4)	0.226	0.21 (0.04-0.99)	0.049	0.32 (0.15-0.68)	0.003	0.53 (0.23-1.21)	0.134	1 (0.27-3.74)	1
ITGA11	0.61 (0.25-1.48)	0.275	0.25 (0.05-1.14)	0.073	0.33 (0.16-0.7)	0.004	0.51 (0.22-1.18)	0.117	1.01 (0.27-3.77)	0.992
GPC4	0.57 (0.23-1.37)	0.208	0.26 (0.06-1.19)	0.083	0.31 (0.14-0.65)	0.002	0.6 (0.27-1.35)	0.216	0.94 (0.25-3.5)	0.921
COL10A1	0.62 (0.25-1.49)	0.283	0.23 (0.05-1.09)	0.064	0.31 (0.15-0.66)	0.002	0.53 (0.23-1.21)	0.13	1.02 (0.27-3.82)	0.979
ANTXR1	0.56 (0.23-1.34)	0.192	0.23 (0.05-1.08)	0.062	0.31 (0.14-0.65)	0.002	0.55 (0.24-1.25)	0.152	0.86 (0.23-3.22)	0.826
SULF1	0.55 (0.23-1.34)	0.189	0.24 (0.05-1.09)	0.065	0.33 (0.16-0.69)	0.003	0.54 (0.24-1.23)	0.141	0.92 (0.25-3.44)	0.906
ARMC7	0.46 (0.19-1.13)	0.092	0.21 (0.05-0.98)	0.047	0.34 (0.16-0.7)	0.004	0.6 (0.27-1.36)	0.221	0.72 (0.19-2.72)	0.632
TMEM14B	0.7 (0.29-1.73)	0.441	0.29 (0.06-1.3)	0.105	0.36 (0.17-0.76)	0.007	0.68 (0.3-1.55)	0.359	0.78 (0.21-2.88)	0.705
AMFR	0.42 (0.17-1.04)	0.06	0.25 (0.06-1.15)	0.076	0.4 (0.19-0.84)	0.016	0.64 (0.28-1.44)	0.278	0.92 (0.25-3.41)	0.899
AFMID	0.36 (0.15-0.91)	0.03	0.27 (0.06-1.26)	0.097	0.3 (0.14-0.64)	0.002	0.52 (0.23-1.18)	0.117	0.77 (0.21-2.87)	0.702
GDAP1	0.46 (0.19-1.11)	0.085	0.23 (0.05-1.06)	0.059	0.37 (0.18-0.77)	0.008	0.62 (0.28-1.41)	0.256	0.79 (0.21-2.94)	0.723

Table S2. Multivariate Cox proportional hazards ratio calculations for *TGFB2* interaction genes with favorable OS outcomes at high TGFB2 and marker gene expression.

	**Gene 2 mRNA**		***TGFB2* mRNA**		**Chemotherapy only**		**Age at Diagnosis**		***TGFB2* by Gene 2 Interaction**	
**Gene 2**	**HR(95% CI)**	***p-val***	**HR(95% CI)**	***p-val***	**HR(95% CI)**	***p-val***	**HR(95% CI)**	***p-val***	**HR(95% CI)**	***p-val***
GDAP1	0.02 (0-0.56)	0.022	0.19 (0.04-0.78)	0.022	1.52 (0.7-3.32)	0.289	1.02 (1-1.04)	0.038	0 (0-0.39)	0.022
TBL1XR1	0.82 (0.57-1.18)	0.289	0.66 (0.42-1.03)	0.067	1.58 (0.73-3.45)	0.247	1.02 (1-1.04)	0.041	1.31 (1.04-1.65)	0.022
RNFT1	0.37 (0.15-0.92)	0.033	0.45 (0.21-0.99)	0.046	1.39 (0.64-3.04)	0.405	1.02 (1-1.04)	0.037	0.16 (0.03-0.79)	0.025
HACL1	0.61 (0.42-0.89)	0.009	0.53 (0.29-0.99)	0.046	1.47 (0.67-3.22)	0.337	1.02 (1-1.05)	0.028	0.54 (0.31-0.97)	0.038
SLC27A2	0.02 (0-0.67)	0.029	0.18 (0.04-0.84)	0.029	1.5 (0.69-3.29)	0.308	1.02 (1-1.04)	0.031	0 (0-0.67)	0.036
NLE1	0.83 (0.59-1.18)	0.3	0.65 (0.41-1.04)	0.071	1.49 (0.68-3.26)	0.323	1.02 (1-1.04)	0.033	0.52 (0.28-0.96)	0.036
TXNDC16	0.96 (0.7-1.31)	0.789	0.7 (0.45-1.1)	0.12	1.59 (0.73-3.5)	0.245	1.02 (1-1.05)	0.023	1.31 (1.09-1.57)	0.004

	**Basal subtype**		**HER2+ subtype**		**Luminal A subtype**		**Luminal B subtype**		**Normal subtype**	
**Gene 2**	**HR(95% CI)**	***p-val***	**HR(95% CI)**	***p-val***	**HR(95% CI)**	***p-val***	**HR(95% CI)**	***p-val***	**HR(95% CI)**	***p-val***
GDAP1	0.46 (0.19-1.11)	0.085	0.23 (0.05-1.06)	0.059	0.37 (0.18-0.77)	0.008	0.62 (0.28-1.41)	0.256	0.79 (0.21-2.94)	0.723
TBL1XR1	0.59 (0.24-1.43)	0.24	0.27 (0.06-1.24)	0.092	0.36 (0.17-0.76)	0.007	0.64 (0.28-1.46)	0.293	0.81 (0.22-3.06)	0.757
RNFT1	0.57 (0.23-1.38)	0.209	0.27 (0.06-1.25)	0.095	0.35 (0.17-0.73)	0.005	0.65 (0.29-1.48)	0.31	0.92 (0.24-3.42)	0.896
HACL1	0.5 (0.2-1.21)	0.123	0.24 (0.05-1.09)	0.064	0.4 (0.19-0.85)	0.017	0.61 (0.27-1.38)	0.239	0.87 (0.23-3.25)	0.836
SLC27A2	0.46 (0.19-1.13)	0.091	0.26 (0.06-1.18)	0.082	0.43 (0.21-0.91)	0.028	0.69 (0.31-1.56)	0.374	0.84 (0.22-3.14)	0.796
NLE1	0.56 (0.23-1.37)	0.205	0.23 (0.05-1.06)	0.06	0.37 (0.17-0.79)	0.01	0.64 (0.28-1.46)	0.287	0.93 (0.24-3.51)	0.911
TXNDC16	0.53 (0.22-1.28)	0.158	0.27 (0.06-1.24)	0.092	0.36 (0.17-0.75)	0.006	0.62 (0.27-1.43)	0.263	0.85 (0.23-3.18)	0.811

Table S3. Kaplan-Meier analysis of *TGFB2*-dependent genes.

Gene 2	Group1	*n*	median OS		Group2	*n*	median OS	Adjusted *p*-val
GDAP1	$TGFB2^{high}/Gene2^{low}$	214	120.0		$TGFB2^{high}/Gene2^{high}$	181	NA	0.0084
GDAP1	$TGFB2^{high}/Gene2^{low}$	214	120.0		$TGFB2^{low}/Gene2^{low}$	180	NA	0.9013
GDAP1	$TGFB2^{high}/Gene2^{low}$	214	120.0		$TGFB2^{low}/Gene2^{high}$	214	NA	0.2875
GDAP1	$TGFB2^{high}/Gene2^{high}$	181	NA		$TGFB2^{low}/Gene2^{low}$	180	NA	0.0084
GDAP1	$TGFB2^{high}/Gene2^{high}$	181	NA		$TGFB2^{low}/Gene2^{high}$	214	NA	0.0710
GDAP1	$TGFB2^{low}/Gene2^{low}$	180	NA		$TGFB2^{low}/Gene2^{high}$	214	NA	0.3784
HACL1	$TGFB2^{high}/Gene2^{low}$	211	114.8		$TGFB2^{high}/Gene2^{high}$	184	NA	0.0188
HACL1	$TGFB2^{high}/Gene2^{low}$	211	114.8		$TGFB2^{low}/Gene2^{low}$	183	NA	0.5859
HACL1	$TGFB2^{high}/Gene2^{low}$	211	114.8		$TGFB2^{low}/Gene2^{high}$	211	NA	0.5859
HACL1	$TGFB2^{high}/Gene2^{high}$	184	NA		$TGFB2^{low}/Gene2^{low}$	183	NA	0.0630
HACL1	$TGFB2^{high}/Gene2^{high}$	184	NA		$TGFB2^{low}/Gene2^{high}$	211	NA	0.0630
HACL1	$TGFB2^{low}/Gene2^{low}$	183	NA		$TGFB2^{low}/Gene2^{high}$	211	NA	0.9499
NLE1	$TGFB2^{high}/Gene2^{low}$	199	120.0		$TGFB2^{high}/Gene2^{high}$	196	NA	0.0308
NLE1	$TGFB2^{high}/Gene2^{low}$	199	120.0		$TGFB2^{low}/Gene2^{low}$	195	NA	0.0942
NLE1	$TGFB2^{high}/Gene2^{low}$	199	120.0		$TGFB2^{low}/Gene2^{high}$	199	NA	0.5486
NLE1	$TGFB2^{high}/Gene2^{high}$	196	NA		$TGFB2^{low}/Gene2^{low}$	195	NA	0.4053
NLE1	$TGFB2^{high}/Gene2^{high}$	196	NA		$TGFB2^{low}/Gene2^{high}$	199	NA	0.0117
NLE1	$TGFB2^{low}/Gene2^{low}$	195	NA		$TGFB2^{low}/Gene2^{high}$	199	NA	0.0397
RNFT1	$TGFB2^{high}/Gene2^{low}$	205	NA		$TGFB2^{high}/Gene2^{high}$	190	NA	0.0164
RNFT1	$TGFB2^{high}/Gene2^{low}$	205	NA		$TGFB2^{low}/Gene2^{low}$	189	NA	0.6066
RNFT1	$TGFB2^{high}/Gene2^{low}$	205	NA		$TGFB2^{low}/Gene2^{high}$	205	NA	0.7300
RNFT1	$TGFB2^{high}/Gene2^{high}$	190	NA		$TGFB2^{low}/Gene2^{low}$	189	NA	0.0761
RNFT1	$TGFB2^{high}/Gene2^{high}$	190	NA		$TGFB2^{low}/Gene2^{high}$	205	NA	0.0392
RNFT1	$TGFB2^{low}/Gene2^{low}$	189	NA		$TGFB2^{low}/Gene2^{high}$	205	NA	0.7300
SLC27A2	$TGFB2^{high}/Gene2^{low}$	218	NA		$TGFB2^{high}/Gene2^{high}$	177	NA	0.0300
SLC27A2	$TGFB2^{high}/Gene2^{low}$	218	NA		$TGFB2^{low}/Gene2^{low}$	176	NA	0.7380
SLC27A2	$TGFB2^{high}/Gene2^{low}$	218	NA		$TGFB2^{low}/Gene2^{high}$	218	NA	0.2235
SLC27A2	$TGFB2^{high}/Gene2^{high}$	177	NA		$TGFB2^{low}/Gene2^{low}$	176	NA	0.0300
SLC27A2	$TGFB2^{high}/Gene2^{high}$	177	NA		$TGFB2^{low}/Gene2^{high}$	218	NA	0.2235
SLC27A2	$TGFB2^{low}/Gene2^{low}$	176	NA		$TGFB2^{low}/Gene2^{high}$	218	NA	0.2235
TBL1XR1	$TGFB2^{high}/Gene2^{low}$	173	114.8		$TGFB2^{high}/Gene2^{high}$	222	NA	0.0121
TBL1XR1	$TGFB2^{high}/Gene2^{low}$	173	114.8		$TGFB2^{low}/Gene2^{low}$	221	NA	0.2887
TBL1XR1	$TGFB2^{high}/Gene2^{low}$	173	114.8		$TGFB2^{low}/Gene2^{high}$	173	NA	0.5042
TBL1XR1	$TGFB2^{high}/Gene2^{high}$	222	NA		$TGFB2^{low}/Gene2^{low}$	221	NA	0.1693
TBL1XR1	$TGFB2^{high}/Gene2^{high}$	222	NA		$TGFB2^{low}/Gene2^{high}$	173	NA	0.0820
TBL1XR1	$TGFB2^{low}/Gene2^{low}$	221	NA		$TGFB2^{low}/Gene2^{high}$	173	NA	0.5042
TXNDC16	$TGFB2^{high}/Gene2^{low}$	210	120.0		$TGFB2^{high}/Gene2^{high}$	185	NA	0.0361
TXNDC16	$TGFB2^{high}/Gene2^{low}$	210	120.0		$TGFB2^{low}/Gene2^{low}$	184	NA	0.8903
TXNDC16	$TGFB2^{high}/Gene2^{low}$	210	120.0		$TGFB2^{low}/Gene2^{high}$	210	NA	0.5136
TXNDC16	$TGFB2^{high}/Gene2^{high}$	185	NA		$TGFB2^{low}/Gene2^{low}$	184	NA	0.0361
TXNDC16	$TGFB2^{high}/Gene2^{high}$	185	NA		$TGFB2^{low}/Gene2^{high}$	210	NA	0.1580
TXNDC16	$TGFB2^{low}/Gene2^{low}$	184	NA		$TGFB2^{low}/Gene2^{high}$	210	NA	0.5337
-	-	-	-	-	-	-	-	-

Table S4. *TGFB2*-independent genes upregulated in tumor tissues.

	**Mean Log_2 TPM ± SEM**		**Fold Increase**	**Linear Contrast**
**Gene**	**Normal**	**Tumor**	**Tumor/Normal**	***p*-value**
ACBD6	4.96 ± 0.03	5.57 ± 0.02	1.52	<0.0001
ARPC5	6.27 ± 0.03	7.18 ± 0.02	1.88	<0.0001
CD276	5.46 ± 0.05	6.52 ± 0.02	2.08	<0.0001
CERCAM	4.66 ± 0.05	5.43 ± 0.03	1.71	<0.0001
CHPF	4.38 ± 0.05	5.87 ± 0.03	2.81	<0.0001
CHPF2	3.74 ± 0.04	4.63 ± 0.02	1.85	<0.0001
COPA	5.51 ± 0.02	6.87 ± 0.03	2.56	<0.0001
CPPED1	2.75 ± 0.04	3.5 ± 0.03	1.69	<0.0001
CTHRC1	4.4 ± 0.08	6.48 ± 0.04	4.25	<0.0001
CYC1	6.26 ± 0.03	6.97 ± 0.03	1.64	<0.0001
DYNLT1	6.33 ± 0.03	7.48 ± 0.02	2.22	<0.0001
ENO1	8.63 ± 0.04	9.2 ± 0.03	1.49	<0.0001
F11R	4.6 ± 0.04	6.38 ± 0.03	3.43	<0.0001
FCGR2A	3.7 ± 0.06	4.64 ± 0.03	1.92	<0.0001
FKBP10	5.59 ± 0.05	6.14 ± 0.03	1.47	<0.0001
GAL3ST4	1.88 ± 0.07	3.18 ± 0.03	2.47	<0.0001
GLRX2	3.41 ± 0.04	4.19 ± 0.02	1.73	<0.0001
ITGB5	6.55 ± 0.04	6.96 ± 0.04	1.32	<0.0001
MMP13	-7.46 ± 0.22	2.23 ± 0.09	830.33	<0.0001
MORC4	3.22 ± 0.03	3.6 ± 0.03	1.30	<0.0001
MRPS14	4.24 ± 0.02	4.92 ± 0.02	1.60	<0.0001
NBDY	4.29 ± 0.03	4.97 ± 0.02	1.61	<0.0001
NCS1	1.89 ± 0.07	3.1 ± 0.04	2.31	<0.0001
PLOD1	5.43 ± 0.03	6.21 ± 0.03	1.72	<0.0001
PLXDC2	3.59 ± 0.05	4.49 ± 0.04	1.87	<0.0001
PPIC	4.86 ± 0.03	5.62 ± 0.03	1.68	<0.0001
PRDX4	6.06 ± 0.03	6.92 ± 0.02	1.81	<0.0001
RASL11B	-0.12 ± 0.11	2.09 ± 0.05	4.65	<0.0001
SDHC	6.76 ± 0.02	7.97 ± 0.02	2.31	<0.0001
SERPINH1	6.47 ± 0.05	7.14 ± 0.03	1.59	<0.0001
SFT2D2	2.79 ± 0.05	3.34 ± 0.03	1.46	<0.0001
SURF4	6.17 ± 0.03	6.85 ± 0.02	1.60	<0.0001
TAGLN2	8.31 ± 0.04	9.29 ± 0.03	1.97	<0.0001
TGFB2	1.54 ± 0.11	2.5 ± 0.06	1.95	<0.0001
TMED9	6.44 ± 0.03	7.63 ± 0.02	2.28	<0.0001
TMPRSS13	0.45 ± 0.2	2.94 ± 0.05	5.62	<0.0001
TP53RK	2.5 ± 0.03	3.71 ± 0.02	2.31	<0.0001
TRPM2	1.56 ± 0.05	3.15 ± 0.04	3.02	<0.0001
TUBA1A	6.59 ± 0.05	7.78 ± 0.03	2.28	<0.0001
UFC1	6.5 ± 0.02	7.61 ± 0.02	2.16	<0.0001
YIPF2	5.17 ± 0.03	5.95 ± 0.02	1.71	<0.0001

Table S5 Multivariate Cox proportional hazards ratio calculations for *TGFB2*-independent genes.

	Gene 2 mRNA		*TGFB2* mRNA		Chemotherapy only		Age at Diagnosis		*TGFB2* by Gene 2 Interaction	
**Gene 2**	**HR(95% CI)**	***p-val***	**HR(95% CI)**	***p-val***	**HR(95% CI)**	***p-val***	**HR(95% CI)**	***p-val***	**HR(95% CI)**	***p-val***
TAGLN2	1.7 (1.34-2.15)	<0.001	0.8 (0.55-1.17)	0.245	1.85 (0.84-4.05)	0.125	1.03 (1.01-1.05)	0.012	0.95 (0.62-1.46)	0.804
PLOD1	1.39 (1.09-1.77)	0.009	0.76 (0.52-1.11)	0.155	1.64 (0.74-3.61)	0.22	1.03 (1.01-1.05)	0.012	0.75 (0.47-1.2)	0.233
ENO1	1.36 (1.07-1.72)	0.013	0.73 (0.5-1.08)	0.114	1.61 (0.73-3.53)	0.235	1.02 (1-1.04)	0.039	0.63 (0.35-1.13)	0.122
TMED9	1.35 (1.07-1.72)	0.012	0.78 (0.53-1.15)	0.209	1.61 (0.73-3.55)	0.234	1.02 (1-1.05)	0.032	0.84 (0.59-1.19)	0.322
GLRX2	1.32 (1.06-1.65)	0.015	0.77 (0.51-1.15)	0.195	1.67 (0.76-3.67)	0.199	1.02 (1-1.05)	0.026	1.03 (0.7-1.51)	0.891
PRDX4	1.32 (1.01-1.74)	0.045	0.77 (0.53-1.14)	0.193	1.71 (0.78-3.73)	0.18	1.03 (1-1.05)	0.022	0.81 (0.51-1.29)	0.378

	Basal subtype		HER2+ subtype		Luminal A subtype		Luminal B subtype		Normal subtype	
**Gene 2**	**HR(95% CI)**	***p-val***	**HR(95% CI)**	***p-val***	**HR(95% CI)**	***p-val***	**HR(95% CI)**	***p-val***	**HR(95% CI)**	***p-val***
TAGLN2	0.26 (0.1-0.68)	0.006	0.18 (0.04-0.86)	0.031	0.41 (0.2-0.87)	0.02	0.75 (0.33-1.69)	0.486	0.93 (0.25-3.48)	0.91
PLOD1	0.34 (0.14-0.85)	0.021	0.29 (0.06-1.3)	0.106	0.41 (0.19-0.87)	0.02	0.78 (0.34-1.8)	0.563	0.88 (0.23-3.31)	0.85
ENO1	0.24 (0.09-0.67)	0.007	0.19 (0.04-0.87)	0.032	0.39 (0.18-0.82)	0.013	0.72 (0.32-1.61)	0.422	0.68 (0.18-2.57)	0.565
TMED9	0.48 (0.2-1.17)	0.107	0.24 (0.05-1.08)	0.062	0.35 (0.17-0.73)	0.006	0.62 (0.28-1.4)	0.252	0.85 (0.23-3.2)	0.809
GLRX2	0.41 (0.16-1.04)	0.061	0.24 (0.05-1.11)	0.069	0.37 (0.18-0.78)	0.009	0.59 (0.26-1.33)	0.201	0.89 (0.24-3.34)	0.863
PRDX4	0.39 (0.16-0.98)	0.045	0.21 (0.05-0.99)	0.048	0.39 (0.18-0.82)	0.013	0.65 (0.29-1.45)	0.289	0.92 (0.25-3.43)	0.905
